# Combined ceftazidime and amikacin resistance among Gram-negative isolates in acute-onset postoperative endophthalmitis: prevalence, antimicrobial susceptibilities, and visual acuity outcome

**DOI:** 10.1186/1869-5760-3-62

**Published:** 2013-10-25

**Authors:** Animesh Jindal, Avinash Pathengay, Manav Khera, Subhadra Jalali, Annie Mathai, Rajeev Reddy Pappuru, Raja Narayanan, Savitri Sharma, Taraprasad Das, Harry W Flynn

**Affiliations:** 1 L V Prasad Eye Institute, GMR Varalakshmi Campus, Visakhapatnam 530040, India; 2 Srimati Kannuri Santhamma Centre for Vitreoretinal Diseases, KAR Campus, LVPEI, KAR Campus, Hyderabad 500034, India; 3 L V Prasad Eye Institutes, Bhubaneswar Eye Institute, Bhubaneswar, Odisha 751024, India; 4 Department of Ophthalmology, Bascom Palmer Eye Institute, University of Miami, Miller School of Medicine, Miami, FL 33136, USA

**Keywords:** Antibiotics, Antibiotic resistance, Endophthalmitis, Gram-negative organisms, Amikacin, Ceftazidime, Vitrectomy

## Abstract

**Background:**

The purpose of this study is to evaluate the prevalence, antimicrobial susceptibilities, and visual acuity outcome of acute-onset postoperative Gram-negative bacterial endophthalmitis cases resistant to both ceftazidime and amikacin seen between 2005 and 2010 at L. V. Prasad Eye Institute, a tertiary care ophthalmic Centre in South India. Medical records of all patients with Gram-negative bacterial endophthalmitis resistant to both amikacin and ceftazidime between 1 January 2005 and 31 December 2010 were reviewed in this non-comparative, consecutive, retrospective case series. Favorable outcome was defined as a best-corrected visual acuity of ≥20/200.

**Results:**

Sixty five (39.6%) of 164 culture-positive postoperative endophthalmitis were caused by Gram-negative organisms. Among these 65 isolates, 32 (49%; 95% confidence interval (CI) 37% to 61%) were resistant to ceftazidime, 17 (26%; 95% CI 15% to 37%) to amikacin, and 12 (18.5%; 95% CI 9% to 27%) to both ceftazidime and amikacin. Eight *Pseudomonas* isolates, three *Enterobacter* isolates, and one *Haemophilus* isolate were resistant to both ceftazidime and amikacin. The isolates were sensitive to fluoroquinolones (42%) and imipenem (50%). Presenting visual acuity was light perception in 10 (83.3%) cases. A final visual acuity ≥20/200 was achieved in 5/12 (41.7%) of these patients.

**Conclusion:**

In the current study, *Pseudomonas* was the most common Gram-negative bacteria resistant to both amikacin and ceftazidime. The emergence of multidrug-resistant bacteria causing endophthalmitis is a matter of concern in India. Alternative antibiotics like imipenem or fluoroquinolones may be considered for the management of these resistant organisms.

## Background

Gram-negative bacteria are less common cause of acute-onset endophthalmitis following cataract surgery [1]. Gram-negative organisms have been isolated in 26% to 42% of patients with cataract surgery related to endophthalmitis in developing countries [1-3] as compared to 5.9% to 12.2% in developed countries [4-12]. The common Gram-negative organisms causing endophthalmitis include species of *Pseudomonas*, *Haemophilus, Klebsiella*, and *Proteus*. Intravitreal ceftazidime or amikacin are commonly used for the empiric treatment of Gram-negative organisms in endophthalmitis. In the Endophthalmitis Vitrectomy Study (EVS), 11% of Gram-negative bacteria were resistant to both amikacin and ceftazidime [5]. The sensitivity of Gram-negative isolates reported from India is 61% to 63% to ceftazidime, 68% to 82% to amikacin, and 73% to 87% to ciprofloxacin [1,2]. Three decades ago (1980 to 1990), the sensitivity of Gram-negative isolates was 98% to amikacin and 100% to ceftazidime [13].

In the current study, the prevalence, antimicrobial susceptibilities, and visual acuity outcome of patients with acute-onset postoperative endophthalmitis is reported for Gram-negative bacteria which were resistant to both ceftazidime and amikacin.

## Methods

Approval was obtained from the local institutional review board and the study followed the Declaration of Helsinki guidelines. Patients with acute-onset postoperative endophthalmitis caused by Gram-negative bacteria occurring between January 2005 and December 2010 and antimicrobial sensitivity data were obtained from the microbiology database. Of these, the isolates resistant to both ceftazidime and amikacin were included in the study and clinical records of these patients were reviewed and analyzed.

All patients were managed by the standard institutional protocol for management of acute endophthalmitis [14]. This essentially consisted of vitreous biopsy or vitrectomy, microscopy, and culture sensitivity of undiluted vitreous, intravitreal antibiotics (vancomycin (1 mg/0.01 ml) + amikacin (400 μg/0.01 ml)/ceftazidime (2.25 mg/0.01 ml)) with or without dexamethasone (400 μg/0.01 ml). Intensive topical antibiotics (ciprofloxacin 0.3% half hourly) and corticosteroid (prednisolone acetate 1%) were administered in all patients. Additional procedures such as repeat intravitreal antibiotics or pars plana vitrectomy/vitreous lavage were performed by the individual treating physicians without a predefined study protocol. Bacterial isolates were identified using Analytical Profile Index (API, Bio Meriux, Craponne, France). The antibiotic sensitivity was checked by the Kirby Bauer disc diffusion method. Anatomical success was defined as the intraocular pressure ≥10 mmHg and best corrected visual acuity ≥20/200.

## Results and discussion

### Results

A total of 510 acute-onset postoperative endophthalmitis patients were identified between 2005 and 2010. Sixty five (39.6%; 95% confidence interval (CI) 32.1% to 47.1%) of 164 culture-positive postoperative endophthalmitis were caused by Gram-negative organisms. Among the 65 Gram-negative cases, 32 (49%; 95% CI 37% to 61%) cases were resistant to ceftazidime, 17 (26%; 95% CI 15% to 37%) were resistant to amikacin, and 12 (18%; 95% CI 9% to 27%) were resistant to both ceftazidime and amikacin.

The mean age of these 12 patients was 61.67 years, ranging from 40 to 85 years. Median time from cataract surgery to onset of symptoms was 7.5 days (range 1 to 14 days). Ten of 12 patients presented with visual acuity of light perception, one had 20/600 and the other 20/80 (Table 1). Ten of 12 patients had hypopyon at presentation and optic disc was visible on indirect ophthalmoscopy in 3 of 12 patients.

**Table 1:** Microbial organisms, interventions, and visual outcome

**S. No.**	**Eye**	**Organism**	**Surgery to symptoms (days)**	**Presenting visual acuity**	**Primary intervention**	**No. of interventions**	**Secondary intervention and intravitreal antibiotics**	**IOL explant**	**Duration of treatment (weeks)**	**Final BCVA**
1	OS	*Pseudomonas aeruginosa*	11	20/600	PPV + IOA	6	C + IOLex	Yes	12	20/100
2	OD	*Pseudomonas aeruginosa*	9	LP	PPV + IOA	1	None	No	8	20/100
3	OD	*Enterobacter*	11	LP	PPV + IOA + IOLex	1	None	Yes	1	LP
4	OS	*Enterobacter*	2	LP	PPV + IOA + IOLex	2	Imip	Yes	16	20/40
5	OD	*Pseudomonas aeruginosa*	5	LP	PPV + IOA + IOLex	2	Imip	Yes	12	20/100
6	OS	*Haemophilus*	1	20/80	PPV + IOA	2	Chlor	No	1	HM+
7	OS	*Pseudomonas aeruginosa*	14	LP	PPV + IOA	2	PPV	No	8	HM+
8	OD	*Enterobacter*	5	LP	PPV + IOA	1	None	No	8	LP
9	OS	*Pseudomonas aeruginosa*	14	LP	PPV + IOA + IOLex	2	C + D	Yes	1	LP
10	OD	*Pseudomonas aeruginosa*	7	LP	PPV + IOA	9	Imip + IOLex	Yes	12	HM+
11	OD	*Pseudomonas aeruginosa*	8	LP	PPV + IOA	2	C + D	No	2	LP
12	OD	*Pseudomonas aeruginosa*	10	LP	PPV + IOA	4	Imip + D	No	16	20/120

In patients with acute postoperative Gram-negative endophthalmitis resistant to both ceftazidime and amikacin. PPV, pars plana vitrectomy; IOA intraocular antibiotic; Imip, imipenem; C, ciprofloxacin; D, dexamathasone; Chlor, chloremphenicol; IOLex, intraocular lens explantation; LP, light perception; HM, hand motion; BCVA, best-corrected visual acuity.

The combined ceftazidime and amikacin resistance was commonly noted in *Pseudomonas aeruginosa* (8/38, 21.05%) followed by *Enterobacter* (3/5, 60%) and *Haemophilus* (1/3, 33.3%) (Table 1). Out of these 12 isolates, five were susceptible to all fluoroquinolones and six were susceptible to imipenem (Table 2). In total, 11 of 12 isolates were susceptible to either of these two drugs. One *Pseudomonas* isolate was resistant to all the tested antimicrobials (Table 2).

**Table 2:** Antibiotic susceptibility in patients with acute postoperative, Gram-negative endophthalmitis resistant to both ceftazidime and amikacin

**No.**	**Organism**	**A**	**Cefa**	**Cefta**	**Chlor**	**Genta**	**Cipro**	**Gati**	**Imip**	**Oflox**	**Pipera**	**Ticar**
1	*Pseudomonas aeruginosa*	R	R	R	R	R	S	S	R	S	-	-
2	*Pseudomonas aeruginosa*	R	R	R	R	R	S	S	R	S	R	R
3	*Enterobacter*	R	R	R	R	R	R	R	S	R	R	R
4	*Enterobacter*	R	R	R	R	R	R	R	S	R	R	R
5	*Pseudomonas aeruginosa*	R	R	R	R	R	R	R	S	R	R	R
6	*Haemophilus*	R	-	R	S	R	S	S	R	S	-	-
7	*Pseudomonas aeruginosa*	R	-	R	R	R	R	R	R	R	R	R
8	*Enterobacter*	R	-	R	R	R	R	R	S	R	R	R
9	*Pseudomonas aeruginosa*	R	-	R	R	R	S	S	R	S	-	-
10	*Pseudomonas aeruginosa*	R	-	R	R	R	R	R	S	R	R	R
11	*Pseudomonas aeruginosa*	R	R	R	R	R	S	S	R	S	-	-
12	*Pseudomonas aeruginosa*	R	R	R	R	R	R	R	S	R	R	R

A, amikacin; Cefa, cefazolin; Cefta, ceftazidime; Chlor, chloramphenicol; Genta, gentamicin; Cipro, ciprofloxacin; Gati, gatifloxacin; Imip, imipenum; Oflox, ofloxacin; Pipera, piperacillin; Ticar, ticarcillin; S, sensitive; R, resistant.

A visual acuity ≥20/200 at last follow-up was achieved in five (41.7%) patients. Of the remaining seven patients with visual acuity <20/200, six went into phthisis and one eye developed thick epiretinal membrane with traction macular detachment. The patient with endophthalmitis caused by *Haemophilus* had a final visual acuity of hand motions despite presenting with visual acuity of 20/80. In six (50%) patients the IOL was explanted, which included four explantations during the primary surgery and in two patients during additional interventions. Nine patients required additional procedures such as repeat intravitreal antibiotic injection with or without vitrectomy (Table 1).

### Discussion

The microbiological spectrum of acute-onset postoperative endophthalmitis from different parts of the world varies significantly. *Staphylococcus* sp. is the most common cause of acute-onset postoperative endophthalmitis following cataract surgery [6-8]. Series from Australia [9,10], North America [6], and Europe [11,12] have reported 6% to 12% Gram-negative bacteria and in the EVS reporting Gram-negative bacteria was isolated in 5.9% (19/323) eyes [5]. The Indian studies, at 26% to 42%, have reported higher incidences of Gram-negative bacterial infection [1,2]. Similarly, in a report from Turkey, a higher rate of Gram-negative bacteria was reported at 35.1% of cases [3].

Current empirical therapies for endophthalmitis generally include vancomycin (1.0 mg/0.1 ml) and ceftazidime (2.25 mg/0.1 ml) or amikacin (0.4 mg/0.1 ml). EVS reported the sensitivity rate of 89.5% for both amikacin and ceftazidime among Gram-negative isolates [5]. Another study from the USA has shown the sensitivity of Gram-negative bacteria to ceftazidime and amikacin at 99% and 100%, respectively [15]. In India, susceptibility of Gram-negative bacteria to amikacin (68% to 82%) and ceftazidime (61% to 63%) is much lower [1,2]. We speculate that widespread use of antibiotics along with cross transfer of multidrug resistance among Gram-negative organisms as a probable cause [16,17].

There may be several mechanisms that contribute to the development of aminoglycoside resistance. These include the deactivation of aminoglycosides by aminoglycoside-modifying enzymes. Other mechanisms include the reduction of the intracellular concentration of aminoglycosides by changes in the outer membrane permeability which is usually a non-specific resistance mechanism, inner membrane transport, active efflux or drug trapping, the alteration of the 30S ribosomal subunit target by mutation, and finally methylation of the aminoglycoside-binding site [18]. Efflux pumps and inhibition of drug intake are common components of multidrug-resistant *Pseudomonas* isolates which prevent accumulation of antibacterial drugs within the bacterium [19].

Ceftazidime is a third-generation cephalosporin and belongs to the beta lactam class of antibiotics. The most common mechanism of resistance to beta lactam antibiotics is by enzymatic deactivation of the drug. Beta lactamase produced by various Gram-negative bacteria renders them inherently resistant to most of the beta lactam antibiotics except third- and fourth-generation cephalosporins. *Pseudomonas* has an additional capability of producing AmpC β-lactamase (also known as cephalosporinase) whose activity is not inhibited by β-lactamase inhibitors including clavulanic acid, sulbactam, and tazobactam [20].

Among the fluoroquinolones, ciprofloxacin is generally the most effective drug against *Pseudomonas*. Other bacteria like *Salmonella*, *Shigella*, *Neisseria*, and *Haemophilus* are highly susceptible to ciprofloxacin regardless of whether the organisms produce β-lactamase or not [21]. The susceptibility profile of ciprofloxacin is reportedly superior to ceftazidime for Gram-negative organisms [1,2,22]. Resistance to fluoroquinolones in general and to ciprofloxacin in particular is reportedly low [23-25]. Also, in our study, ciprofloxacin was the drug of choice against Gram-negative bacteria [26]. In our database, out of the 65 cases of Gram-negative bacterial postoperative endophthalmitis, 77% isolates were sensitive to ciprofloxacin, and in the current series, nearly 42% of Gram-negative bacteria that were resistant to both ceftazidime and amikacin showed susceptibility to ciprofloxacin. Since the elimination half-life of intravitreal ciprofloxacin is short [27], oral administration may be considered in such patients. The intravitreal concentration of ciprofloxacin after oral administration has been demonstrated to be above the minimum inhibitory concentration (MIC-90, i.e., minimum antibiotic concentration inhibiting 90% of strains) of most of the organisms in inflamed eyes [28].

In the current study, 50% of the Gram-negative isolates which were resistant to both amikacin and ceftazidime were sensitive to imipenem. Imipenem has a broad spectrum of activity against both aerobic and anaerobic and Gram-positive and Gram-negative bacteria including *Pseudomonas* and *Enterococcus* species. It acts by inhibiting cell wall synthesis of various Gram-positive and Gram-negative bacteria [29]. It is stable to hydrolysis by the common plasmid-mediated beta-lactamases produced by various bacteria and lacks cross resistance with penicillins and third-generation cephalosporins [30]. Intravitreal imipenem may limit intraocular inflammation and retinal tissue damage when given early in the course of *Pseudomonas* endophthalmitis [31]. It is generally nontoxic in animal models at concentrations that are far higher than the MIC 90 of 3.6 to 12.5 μg/ml against *Pseudomonas* infection and may offer promise in the treatment of endophthalmitis after intraocular surgery or perforating eye injuries [32].

In the EVS, 56% of eyes infected with Gram-negative bacteria regained 20/100 [4]. Multidrug resistance is more common in Gram-negative bacteria (78.6%) compared to Gram-positive bacteria (21.4%) [33]. The multidrug resistance and higher number of *Pseudomonas* in the current study may explain the poorer outcome in this study as compared to EVS.

There are certain limitations in this retrospective study. Firstly, antibiotic susceptibility in this study was tested by disc diffusion method and was not confirmed by MIC. Secondly, the number of cases included in the study is relatively small.

## Conclusions

In conclusion, resistance to both amikacin and ceftazidime among Gram-negative isolates was not uncommon in the current study. *Pseudomonas* was the most common multidrug-resistant Gram-negative bacteria isolated and the visual acuity outcomes were generally poor. An alternative group of drugs like ciprofloxacin or imipenem may be considered for the management of these cases. Drug resistance is an emerging problem among Gram-negative isolates causing acute-onset postoperative endophthalmitis in India.

## Competing interests

The authors declare that they have no competing interests.

## Authors’ contributions

AJ and MK carried out the data collection and data analysis and drafted the manuscript. AP is one of the treating physician and also carried out the correction of the manuscript. SJ, AM, RRP, RN, and TD are the other treating physicians. SS is the microbiologist. HWFJ corrected the manuscript. All authors read and approved the final manuscript.
